# Strategies for the efficient use of diagnostic resource under constraints: a model-based study on overflow of patients and insufficient diagnostic kits

**DOI:** 10.1038/s41598-020-77468-2

**Published:** 2020-11-26

**Authors:** Naoshi Tsuchida, Fumihiko Nakamura, Kazunori Matsuda, Takafumi Saikawa, Takashi Okumura

**Affiliations:** 1 School of Medicine, Hokkaido University, Sapporo, 060-8638 Japan; 2 Kitami Institute of Technology, Kitami, 090-8507 Japan; 3 Graduate School of Mathematics, Nagoya University, Nagoya, 464-0814 Japan

**Keywords:** Public health, Statistics

## Abstract

This article addresses an optimisation problem of distributing rapid diagnostic kits among patients when the demands far surpass the supplies. This problem has not been given much attention in the field, and therefore, this article aims to provide a preliminary result in this problem domain. First, we describe the problem and define the goal of the optimisation by introducing an evaluation metric that measures the efficiency of the distribution strategies. Then, we propose two simple strategies, and a strategy that incorporates a prediction of patients’ visits utilising a standard epidemic model. The strategies were evaluated using the metric, with past statistics in Kitami City, Hokkaido, Japan, and the prediction-based strategy outperformed the other distribution strategies. We discuss the properties of the strategies and the limitations of the proposed approach. Although the problem must be generalised before the actual deployment of the suggested strategy, the preliminary result is promising in its ability to address the shortage of diagnostic capacity currently observed worldwide because of the ongoing coronavirus disease pandemic.

## Introduction

In the ongoing pandemic caused by the novel coronavirus disease called as COVID-19, the scarcity of diagnostic resources has been a serious constraint in the pandemic response^1,2^. COVID-19 is confirmed by reverse transcription polymerase chain reaction (RT-PCR) technique, whose test capacity is limited compared with rapid diagnostic kits that are universally used for influenza^3^. However, this type of shortage may occur even in seasonal influenza, in special occasions. For example, a shortage may occur even at an ordinary medical institution when there is a surge of patients by an uncontrolled endemic or delivery of additional supplies is delayed. The supply could also be limited due to the damage of the supply chain of factory production by accidents or disasters.

We encountered one such special situation in which there is a shortage of rapid diagnostic kits for seasonal influenza. In the university where one of the authors works, the Health Administration Center orders rapid diagnostic kits for influenza at the beginning of an epidemic season (usually from December to March in Japan). However, the university clinic is running with its constant operational budget and, thus, cannot order the kits for all patients who are suspected of influenza. This was partly because the clinic offers medical services for the university community as a fringe benefit and does not charge these users even for the cost of medical supplies. Accordingly, there emerges a situation where the scarcity of diagnostic resources constraints the operation, and there must be a mechanism to allocate the limited resources among patients in an optimised manner.

This type of situation rarely occurs at ordinary medical institutions because they can place additional orders when their stocks run out or may have excess stocks because they are gaining revenue from patients. Moreover, the diagnosis of influenza itself can be made clinically. However, we assume that the use of rapid kits is beneficial in objectively assessing the diagnostic accuracy. Because subtypes of viruses may change in mid-season, which might deteriorate clinicians’ diagnostic accuracy, we needed an approach to distribute a limited set of diagnostic kits throughout the season. Accordingly, to avoid providing scarce resources in a first-come first-served basis, research efforts must address the optimal distribution of the limited diagnostic resources.

In previous studies concerning the scarcity of medical resources for infectious agents, the distribution of preventive vaccines and remedial resources, such as drugs and ventilators, has been mostly discussed^4,5^. However, this study discusses the problem of the distribution of rapid diagnostic kits among patients in a situation where the demands are expected to surpass the supplies in a single epidemic season. This type of problem has not gained much attention in the field, and thus, this study applied the theoretical approach first, focusing on the mathematical aspect of the resource distribution problem. To this end, we abstracted the problem as a theoretical study and simplified the model, leaving clinical details of the examination and epidemiological impact on the target population for our future work.

The primary goal of optimisation in this study is to randomly distribute the diagnostic kits among patients in a time series and assess physicians’ diagnostic accuracy, in some special occasions. This study addresses the inequality of diagnostic resources and does not deal with virological surveillance. In “Method” section, we discuss the characteristics of the problem and propose several possible strategies. The distribution problem of diagnostic resources differs from the traditional optimisation problem of vaccination rationing, in the choice of the objective functions that are to be optimised. While the vaccination problem targets the minimisation of the total number of patients or death toll^4,6,7^, we aimed to create a metric to evaluate the distribution strategies for diagnostic resources. “Results” section presents the simulation results and algorithms evaluated according to the metric. This is followed by a discussion in “Discussion” section and conclusions in “Conclusion” section. Clinical implications and implementations of the proposed approach are summarised in the concluding remarks.

## Methods

### Problem setting

In this study, we evaluated the problem of distribution of diagnostic kits among patients who need to be tested, where the number of patients surpasses the available stock of kits. To provide a preliminary result in this problem domain for future references, we simplified the problem as follows. Some of the assumptions may seem unrealistic because of the nature of the theoretical study. The assumptions and constraints will be relaxed in the future work, and this decision is discussed in “Discussion” section.

- The university clinic examines patients and makes a diagnosis based on clinical findings. Due to budgetary constraints, the clinic cannot afford a diagnostic kit for every suspected patient.
- The initial number of rapid diagnostic kits is provided. The clinic consumes one rapid diagnostic kit to diagnose a suspected patient, and the number of kits decreases as the medical institution consumes it. The initial number of diagnostic kits is set to be smaller than the expected number of suspected patients. Therefore, the kits are expected to run out, which is our main interest.
- We do not consider an additional supply of kits. If the supply capacity during the season is predictable, the problem becomes the same with the case of no supply just by adding the supply number to the initial number. Moreover, we do not assume expiration of the kits. Although they might seem unrealistic, it is the constraint the university clinic actually has, as described.
- The analysis is performed for an entire epidemic season, only with one peak in the number of patient visits. This constraint will be relaxed in the future study.
- It is desirable that all kits have been used at the end of one epidemic season. Conversely, at any moment in the season, the number of kits should be kept positive to accommodate new patients.
- In the clinic, we make referrals to nearby medical institutions, once diagnosis is made, because the university health center does not provide therapeutic care of influenza for budgetary reasons. We further simplify the problem by assuming that each patient visits the clinic only once and does not revisit.

When the expected number of patients surpasses the stock of the kits, the simplest strategy is to use the kits on a first-come first-served basis. However, this strategy exhausts the reserve and does not meet the defined goal. Accordingly, the distribution must be *probabilistic*, at a rate less than one per suspected patient. We call this the consumption rate, which is a variable reflecting the uncertainty of the number of patients in the future. For example, if too many kits are used at a high consumption rate in the early days of an epidemic season, the clinic will run short of stock and be forced to lower the consumption rate in the subsequent days of the season.

An ideal consumption rate would be constant throughout an epidemic season, such that it prevents shortage of kits during the season and kits remaining at the end of the season. Additionally, a constant rate enables the unbiased and uniform sampling of patients, which results in more accurate statistics for disease incidence. Moreover, from the patient’s point of view, a constant consumption rate is preferable because it gives each patient an equal probability of being diagnosed objectively using the kit (i.e. distributive justice).

Forecasting the epidemic curve (time series of the number of the patients), a major issue in theoretical epidemiology, is the key to such a strategy. Several models on the nature of epidemic/pandemic spread have been proposed^8^. In this study, since we focus on diagnostic kit shortage, it is important to forecast the scale of the epidemic. Therefore, we adopt a simple susceptible–infected–recovered (SIR) model, which meets the objective (Appendix A.1). The SIR model is so simple that it ignores some aspects of an epidemic. Especially, since it originally focuses on the nationwide spread, the SIR does not consider the population mitigation or inter-regional effect of infection, which is important in the regional analysis. However, it can describe the two different stages of an epidemic season, i.e., the early stage when the patients number increases and the peak stage when the number of new cases reaches the maximum, under simple assumptions. For these reasons, we consider that the SIR model is sufficient for our purpose of this study. In Appendix A.3, we show that the SIR model well describes our data.

### Data

The data we utilised were the weekly statistics of influenza incidence in Kitami City, Hokkaido, Japan. The number included newly confirmed influenza cases at medical institutions (hospitals and clinics) located in Kitami. The data consisted of $N = 38$ medical institutions, and $T=25$ weeks in the 2018 epidemic season (from 5 November 2018 until 28 April 2019) and 21 weeks in the 2017 epidemic season (from 27 November 2017 until 22 April 2018).

The medical institutions were categorized into three groups, A, B and C, by descending order of the number of patients during a single epidemic season. Table 1 shows the mean, standard deviation, and maximum and minimum values of the total number of patients for each group and for all the medical institutions.

**Table 1:** The mean, standard deviation, maximum and minimum values of the total number of patients for each group and for all the institutions.

Group	N	2018				2017			
		Mean	SD	Max	Min	Mean	SD	Max	Min
A	13	428.2	211.3	827	191	516.5	278.4	1001	243
B	13	168.5	37.0	239	99	181.0	32.3	239	127
C	12	74.3	31.3	121	26	82.3	30.7	125	37
Total	38	227.6	195.5	827	26	264.6	247.6	1001	37

### Evaluation metric

To establish an evaluation metric, we mathematically described the problem in “Problem setting” section using the following notations:

- *N* : the number of medical institutions. In this paper, $N=38$.
- *T* : the length of the epidemic season measured in weeks, where $T = 25$ for 2018 and $T = 21$ for 2017. Note that the exact value of *T* is not known until the epidemic season ends, but the value can be estimated from past epidemic patterns.
- $P^{R}_t$ : the observed number of new suspected patients during the *t*-th week for $t=1,\ldots ,T$.
- *X* : a strategy for how to determine the consumption rate during each week. As we discuss later in this paper, we introduced three strategies $X=M$, $S_1$, and $S_2$ and used one “ideal” distribution *I* as a benchmark.
- $W^{X}_{t}$ : the number of unused kits in the *t*-th week with strategy *X*. Note that $W^{X}_{t} - W^{X}_{t-1}$ is the number of kits used during the *t*-th week.
- $r^{X}_{t}$ : the consumption rate (the probability of whether a kit is used for a patient with suspected influenza) in the *t*-th week with strategy *X*.
- $W_{{\mathrm {init}}}$ : the initial number of unused kits. Note that $W_5^X = W_{{\mathrm {init}}}$ since the strategies are defined from the sixth week for comparison purposes. This is because strategy *M* requires the data from the initial five weeks to predict the number of patients.

As noted in “Problem setting” section, it is ideal if $r^{X}_{t}$ is constant. The strategy closest to this ideal situation is achieved by setting $r^{X}_{t}$ as the ratio between the number of unused kits in the *t*-th week and the total number of patients to be tested, from the $(t + 1)$-th week to the *T*-th week, as follows:

**Strategy** *I*  (ideal distribution). *We define the “ideal distribution”, denoted by* $X=I$, *by setting the number of unused kits recursively in each week as*

1 $$\begin{aligned} W^{I}_{5} = W_{{\mathrm {init}}},\quad r^{I}_{t} := \min \left\{ \frac{W^{I}_{t}}{ \sum _{s=t + 1}^T P^{R}_{s}}, 1\right\} ,\quad W^{I}_{t + 1} := \max \{ W^{I}_{t} - \lfloor r^{I}_{t} \cdot P^{R}_{t}\rfloor ,\ 0 \}, \end{aligned}$$

*where* $\lfloor x \rfloor$ *is the integer part of* *x*, *called the floor function. Note that the observed value of* $r^{I}_{t}$ *is not constant because of the effect of truncation to the integer.*

Now, we define the following *loss function* $E^{X}$ as an evaluation metric, which measures the difference between the ideal distribution $X = I$ and strategy *X*.

2 $$\begin{aligned} \displaystyle E^{X} := \frac{1}{(T - 5)\cdot W_{{\mathrm {init}}}} \sum _{t=6}^T |W^{X}_{t} - W^{I}_{t}|. \end{aligned}$$

Note that the summation begins from the sixth week as the strategies are defined from that week. The value of function $E^X$ is zero when the strategy is ideal ($X=I$), and increases up to 1/2 as the strategy deviates from the ideal strategy. Using too many kits during the early period will lead to a shortage of kits during the later period and result in a large $E^X$value. On the other hand, keeping too many kits during the early period leaves too many unused kits at the end, which also results in a large $E^X$ value. By minimising loss, the risk of excess use or reserving too many unused kits during the epidemic season can be minimised. This strategy of minimising risk is *efficient*; therefore, the most efficient strategy is obtained by minimising the loss value.

### Prediction-based strategy

In this section, we propose a strategy to consume the diagnostic kits, based on a prediction of patients’ visits. First, we set the following notation:

- $\hat{P}_{t}(s)$: the predicted number of patients during the *t*-th week for $t=s + 1,\ldots ,T$ calculated by the parameters estimated from {$P^{R}_1, \ldots , P^{R}_s$}.

To predict the values of $\hat{P}_{t}(s)$, we utilised the SIR model, which is one of the simplest models for epidemics^9–11^.

**Figure 1:**
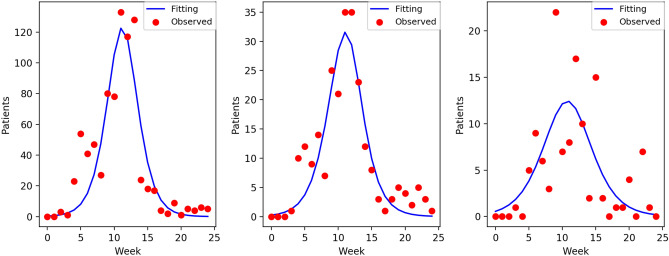
The observed number of patients in each week and the fitting curves for them using an SIR model for institutions in group A (left), group B (middle) and group C (right).

We used the following fitting function to estimate the number of predicted patients $\hat{P}_{t}(s)$: 
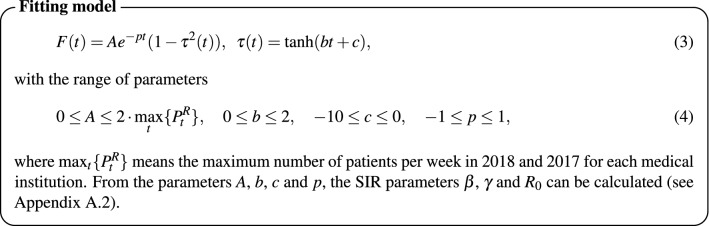


First, we fitted the parameters of each individual medical institution using the number of patients throughout the season. Then, we determined the parameter ranges shown in Eq. (4) to cover the parameters of all the institutions. Figure 1 displays the observed number of patients during each week and the fitting curve using the SIR model. In Table 2, we summarised the mean, maximum and minimum values of parameters *A*, *b*, *c*, *p*, and $R_0$ obtained using the SIR fitting for each group.

**Table 2:** The mean, maximum and minimum values of parameters *A*, *b*, *c* and *p* in the SIR model by groups A, B and C. The values are calculated by fitting to the total number of patients of each medical institution in 2018 and 2017.

	*A*			*b*			*c*			$^{\mathrm {p}}$		
Group	Mean	Max	Min	Mean	Max	Min	Mean	Max	Min	Mean	Max	Min
**2018**												
A	61.6	124.4	22.6	0.39	0.91	0.20	− 4.30	− 2.07	− 10.0	0.50	1.00	− 0.26
B	27.8	58.5	13.7	0.57	1.39	0.21	− 5.18	− 2.58	− 10.0	0.21	1.00	− 1.00
C	8.6	17.6	1.6	0.87	2.00	0.18	− 5.69	− 2.23	− 10.0	0.12	1.00	− 1.00
Total	33.3	124.4	1.6	0.60	2.00	0.18	− 5.04	− 2.07	− 10.0	0.28	1.00	− 1.00
**2017**												
A	38.5	149.0	2.9	0.40	0.98	0.19	− 3.77	− 2.06	− 9.27	0.84	1.00	0.51
B	29.2	106.5	5.4	0.30	0.63	0.17	− 2.94	− 1.16	− 5.40	0.21	0.48	0.00
C	32.4	128.2	6.6	0.82	2.00	0.19	− 6.27	− 1.72	− 10.0	− 0.67	0.00	− 1.00
Total	33.4	149	2.9	0.50	2.00	0.17	− 4.28	− 1.16	− 10.0	0.15	1.00	− 1.00

**Figure 2:**
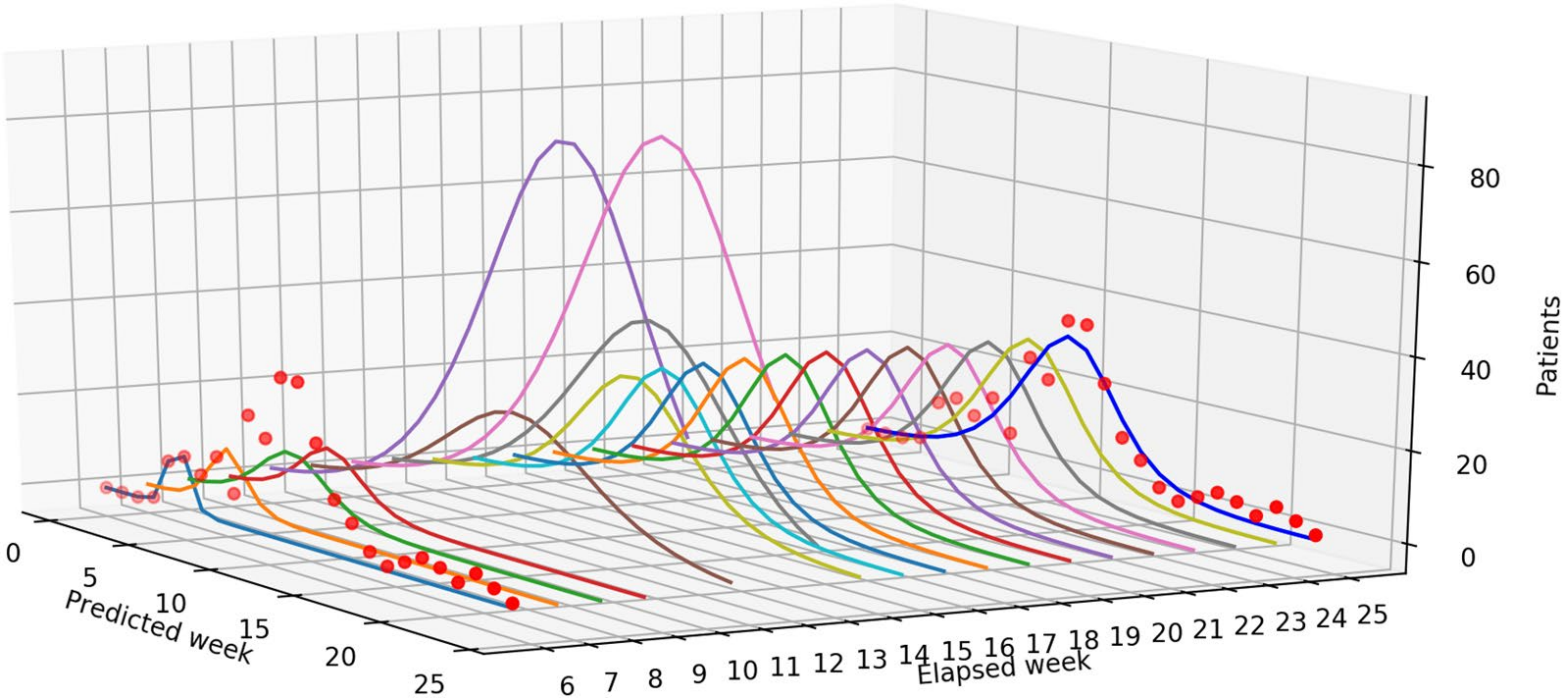
Fitting curves at each week for one representative medical institution in group B in 2018. Each curve represents each *t*-th week ($t=6, 7, \dots , 25$) up to which the fitting is based on the data, shown in the horizontal ‘Elapsed week’ axis. The horizontal ‘Predicted week’ represents the week when it is predicted that the number of patients will be observed. The dots are the observed numbers of patients (two series at $t = 6$ and $t = 25$ are the same).

Next, within the parameter ranges shown in Eq. (4), we calculated the parameters at the *s*-th week using the data up to that week, and estimated the number of patients in the future, that is, the value of $\hat{P}_{t}(s)$, where $t=s+1,\ldots ,T$. An example of $\hat{P}_{t}(s)$ is shown in Fig. 2. The multiple curves seen in the figure show the different weeks until the prediction based on the data. Note that as we need some data to start fitting, we started the prediction estimation from the sixth week. In this example, the observed numbers exhibited only one major peak, and the series of curves converged to a fine approximation as the weeks went on.

Now, utilising prediction $\hat{P}_{t}(s)$, we defined our main strategy, as follows.

**Strategy** *M* (main strategy).  *For our main strategy, we set the kit usage rate in the* *t-th week* $r^{M}_{t}$ *as the ratio between the number of unused kits at the* *t-th week and the total of the predicted number of patients from the* $(t+1)$*-th week to the* *T-th week; namely, the number of unused kits in each week was determined recursively on* *t*, *as follows:*

5 $$\begin{aligned} W^{M}_{5} = W_{{\mathrm {init}}},\quad r^{M}_{t} := \min \left\{ \frac{W^{M}_{t}}{\sum _{s=t + 1}^T \hat{P}_{s}(t)}, 1\right\} , \quad W^{M}_{t + 1} := \max \{ W^{M}_{t} - \lfloor r^{M}_{t} \cdot P^{R}_{t}\rfloor ,\ 0 \}. \end{aligned}$$

Strategy *M* is equivalent to the ideal strategy *I*, if the predicted variable $\hat{P}_{s}(t)$ matches $P^{R}_{s}$, that is, $\hat{P}_{s}(t)=P^{R}_{s}$.

### Alternative strategies

To clarify the characteristics of our proposed strategy *M*, we introduced alternative strategies that do not depend on a prediction of patients’ visits $\hat{P}_{t}(s)$.

**Strategy** $S_1$ (simple strategy 1). *For this strategy, we can use half the number of unused kits in each week; namely, the number of unused kits during each week is determined recursively on* *t*, *as follows:*

6 $$\begin{aligned} W^{S_{1}}_{5} = W_{{\mathrm {init}}},\quad W^{S_{1}}_{t + 1} := W^{S_{1}}_{t} - \min \left\{ \left\lfloor \frac{W^{S_{1}}_{t}}{2}\right\rfloor ,\ P^{R}_{t} \right\} ,\quad r_t^{S_1}=\frac{W_{t}^{S_1}-W_{t+1}^{S_1}}{P_t^{R}}. \end{aligned}$$

**Strategy** $S_2$ (simple strategy 2). *For this strategy, the usage of kits in each week is determined by the number of the rest of the weeks; namely, the number of unused kits in each week is determined recursively on* *t* *as below. Note that we supposed that the value of* *T* *can be estimated at the beginning of an epidemic season.*

7 $$\begin{aligned} W^{S_{2}}_{5} = W_{{\mathrm {init}}},\quad W^{S_{2}}_{t + 1} := W^{S_{2}}_{t} - \min \left\{ \left\lfloor \frac{W^{S_{2}}_{t}}{T - t}\right\rfloor ,\ P^{R}_{t} \right\} ,\quad r_t^{S_2}=\frac{W_{t}^{S_2}-W_{t+1}^{S_2}}{P_t^{R}}. \end{aligned}$$

**Figure 3:**
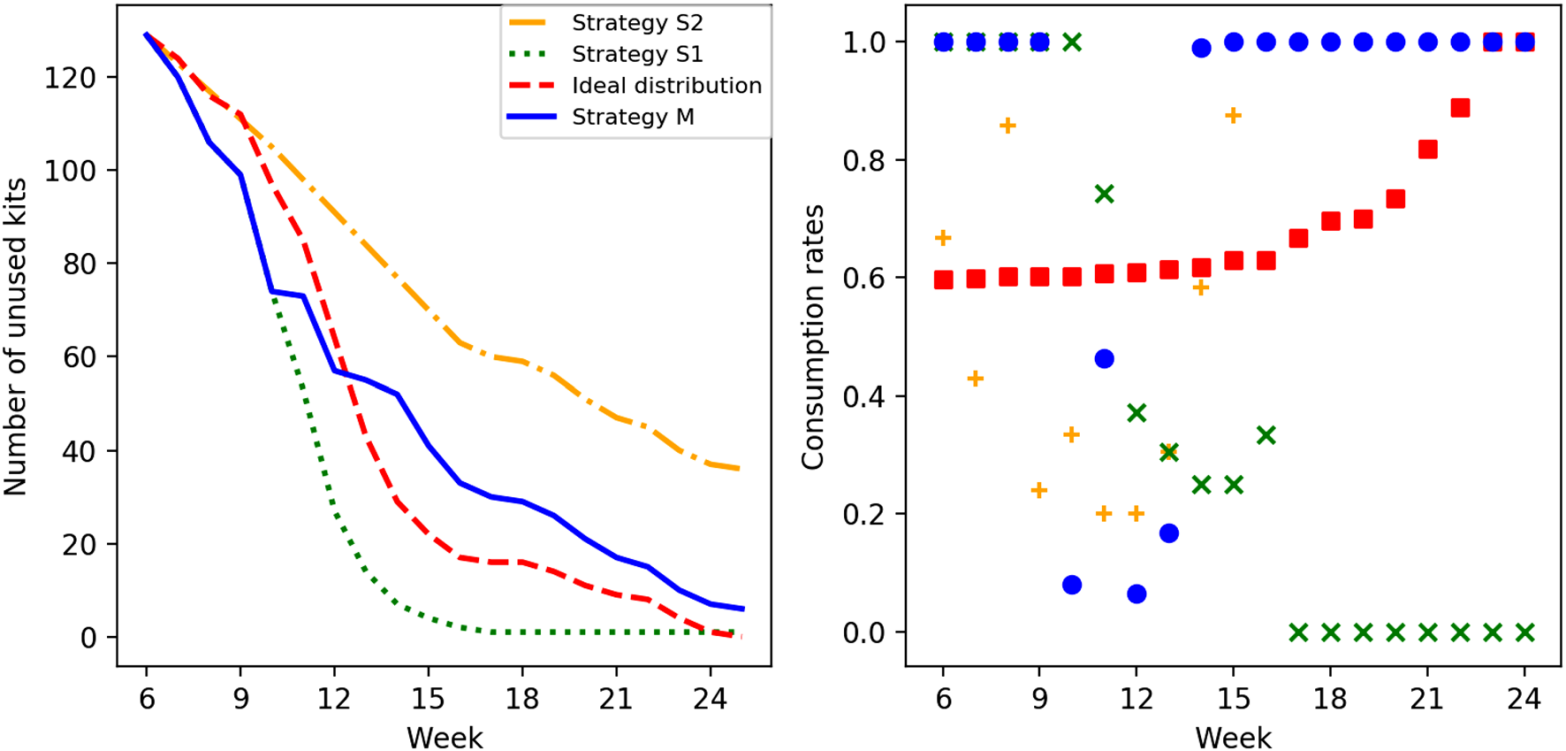
Left: The number of unused kits for each week. Right: An example of the consumption rate $r^X_t$. These charts are for the representative medical institution belonging to group B, which is shown in Fig. 2. The green cross ($\times$) and dotted line stand for strategy $S_1$, the yellow plus ($+$) and dash–dotted line for strategy $S_2$, the blue bullet ($\bullet$) and solid line for strategy *M*, and the red square ($\blacksquare$) and dashed line for strategy *I* (note that this should be constant in theory, but it is not and increases due to the truncation effect).

Figure 3 shows the basic characters of the three strategies (our main strategy *M* and two alternative strategies $S_1$ and $S_2$) and the ideal distribution (*I*). The weekly development of the consumption rates and the number of unused kits were plotted. Intuitively, the strategy was considered better if the corresponding curves were closer to those of the ideal distribution (strategy *I*).

## Results

As noted in “Introduction” section, we considered the shortage of diagnostic kits, in which the initial number of kits $W_{{\mathrm {init}}}$ was insufficient when compared to the number of total patients $\sum _i P_i^R$. To describe the severity of the shortage, we defined the *filling rate* as the ratio $f=W_{{\mathrm {init}}}/\sum _i P_i^R$, that is, the ratio between the initial number of kits and the total number of patients. The value of *f* is 1 when there are just enough kits to cover all the patients, and decreases from 1 as the shortage grows.

With the ideal distribution $X=I$ and $r_t=r_t^I$, the required reserve of unused kits is kept throughout the season and no unused kits remain at the end of the season. In reality, the number of patients $P_i^R$ fluctuates with uncertainty during the season, and the total number cannot be determined before the end of the epidemic season. Therefore, the ideal distribution is not feasible, and we need to consider distribution strategy *X*. To measure the efficiency of a strategy, we introduced the loss function $E^X$ in Eq. (2).

**Figure 4:**
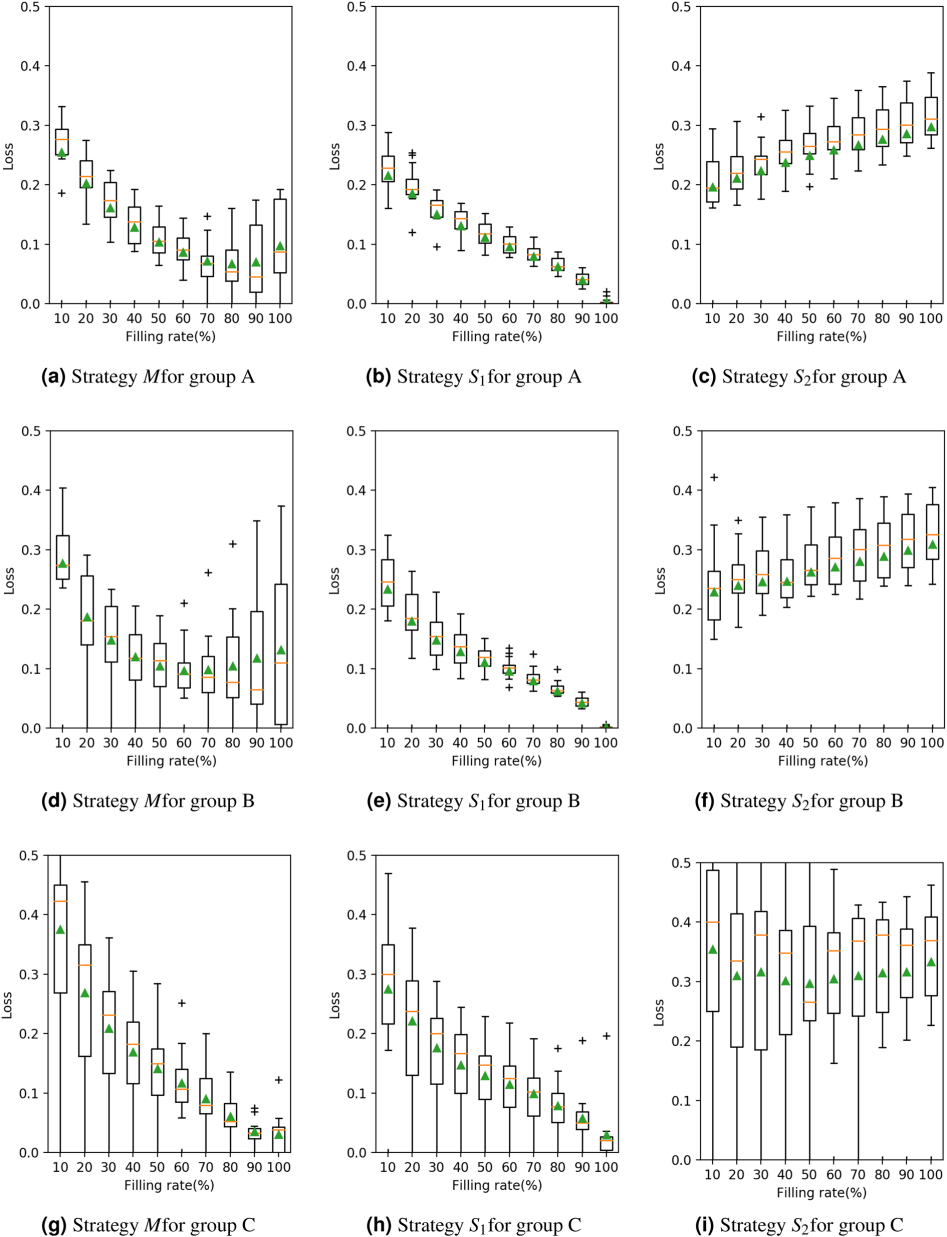
The relationship between the loss value (vertical) and the filling rate (horizontal) for each group (A, B, C) and strategy (*M*, $S_1$, $S_2$) in 2018. The triangle symbol ($\blacktriangle$) denotes the mean. The box–bar plot shows the median, minimum, maximum and quartiles. The plus symbol ($+$) denotes the outlier points.

**Figure 5:**
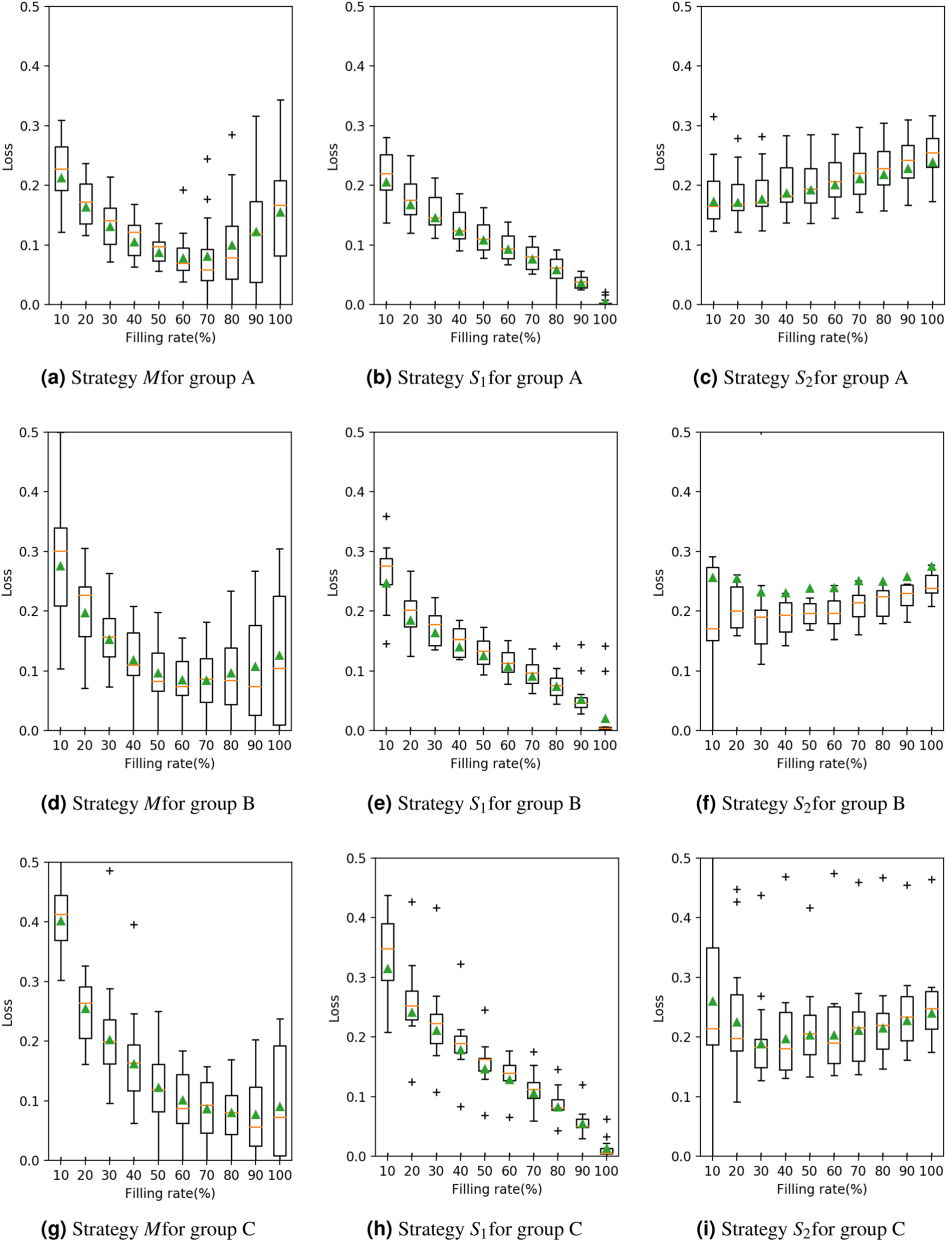
The relationship between the loss value (vertical) and the filling rate (horizontal) for each group (A, B, C) and strategy (*M*, $S_1$, $S_2$) in 2017. The triangle symbol ($\blacktriangle$) denotes the mean. The box–bar plot shows the median, minimum, maximum and quartiles. The plus symbol ($+$) denotes the outlier points.

Figures 4 and 5 represent the relationship between the loss value $E^X$ and the filling rate *f* in 2018 and 2017, respectively. These figures are shown separately for each group and strategy, and each figure describes the mean value and other statistics of $E^X$. In these figures, a lower curve means a smaller loss value; hence, the corresponding strategy is better. Strategy $S_2$, which has larger loss values and a higher curve, is worse than the other strategies.

When comparing strategies *M* and $S_1$, two features are observed. First, *M* and $S_1$ have a similar level of loss value. Second, the loss value is decreasing monotonously and the minimum is achieved at $f\simeq 1$, in strategy $S_1$, while the curve has a minimum at $f < 1$ in strategy *M*. Second, strategy $S_1$ easily faces a kit shortage before the end of the period under condition $f < 1$. As we are trying to avoid this situation, this is not a desirable property. These results suggest that the strategies with prediction (*M*) are more reasonable than those without prediction ($S_1$ and $S_2$).

**Figure 6:**
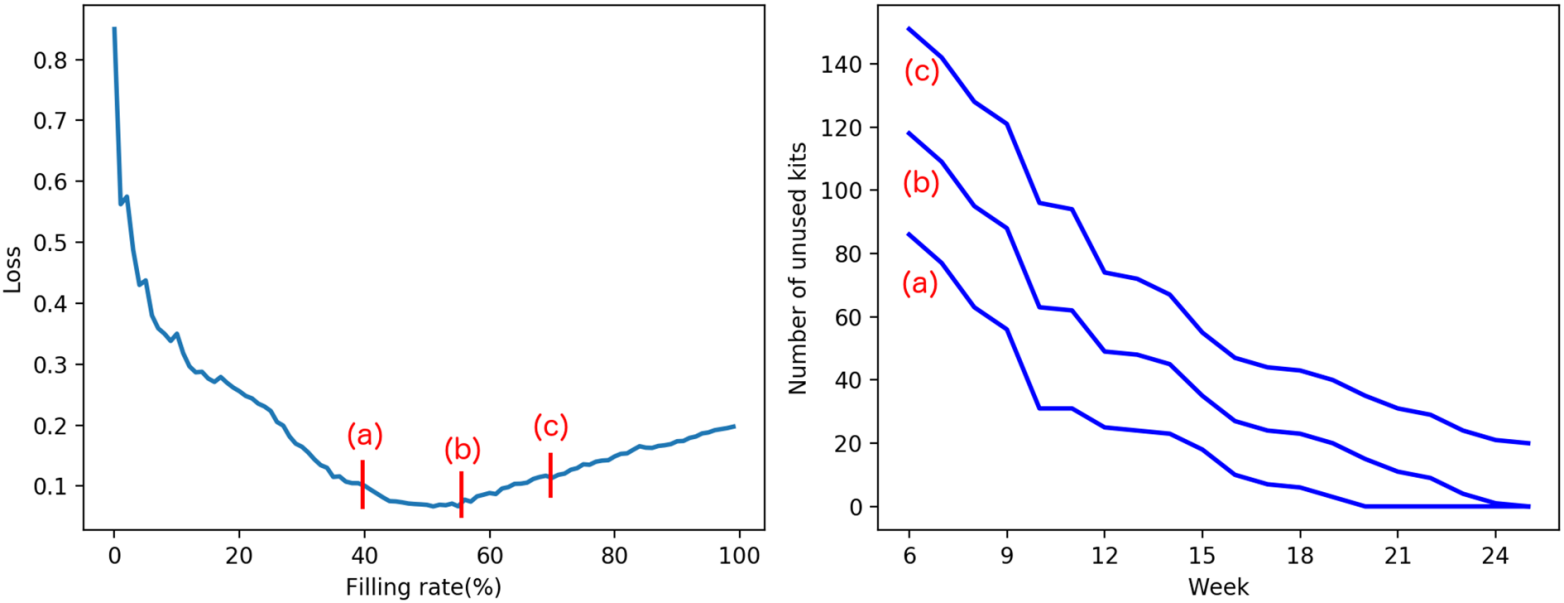
Left: The relationship between the filling rate and the loss for one representative medical institution in group B when strategy *M* is applied. Right: The time series of the number of unused kits for the filling rates of (**a**) 40%, (**b**) 55% (giving the minimum loss value) and (**c**) 70%.

Figure 6 shows the details of how the level of the filling rate *f* affects the loss when strategy *M* is applied to one representative medical institution belonging to group B. The left subfigure shows the relationship between the loss value and the filling rate, and we can see that a 60% filling rate results in a minimum loss value for this institution. The right subfigure shows the time series of the number of unused kits depending on the filling rate of (a) 40%, (b) 55% and (c) 70%.

Figure 6 shows that the unused kits are exhausted before the end of the season in the case of (a). As discussed above, this situation should be avoided. Conversely, a remarkable number of kits remains unused at the end of the season in the case of (c), which should also be avoided as the kits left behind become waste in our problem setting. In the case of (b), no shortage or excess of unused kits was observed. In other words, if the minimum loss value is achieved at $f < 1$, then we can minimize the risk of excess or shortage when compared with the case $f = 1$.

**Figure 7:**
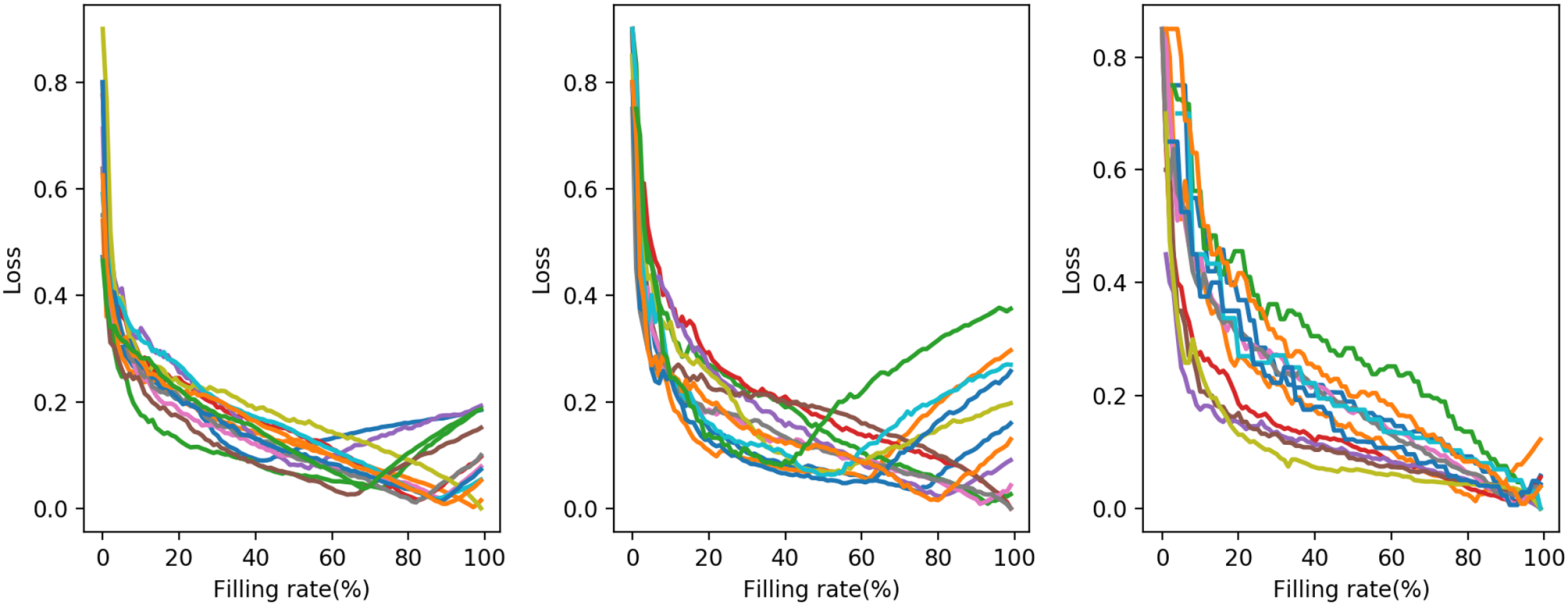
The relationship between the loss value (vertical) and the filling rate (horizontal) for each medical institution shown by a line when we apply strategy *M* to the 2018 data. Left: institutions belonging to group A; middle: those in group B; and right: those in group C.

To investigate the difference across the medical institutions, Figure 7 shows the loss values for each individual medical institution with strategy *M* in 2018. Each line shows an individual medical institution. Two patterns of lines are observed when we focus on when the minimum loss value is achieved. One pattern shows that the minimum loss value is achieved at the filling rate of less than 1 and the other pattern shows that the minimum loss value is achieved close to 1. In the former pattern, strategy *M* is more efficient than the others, and this pattern is observed mainly in large medical institutions belonging to groups A and B. In the latter pattern, strategy *M* cannot reduce the shortage or excess risk, and this situation is seen mainly in smaller institutions belonging to group C. The results suggest that for strategy *M* to be functional, it is important to know the conditions that minimise the loss function at $f < 1$.

## Discussion

In summary, by estimating the number of patients based on the SIR model, strategy *M* can examine the patients more efficiently than strategies $S_1$ and $S_2$, in the sense that

1. the distribution is close to the ideal distribution (the loss value is close to zero) and
2. there is a lower risk of a shortage or an excess of kits during the period (there is a minimum value under the filling rate less than 1).

It is worth noting that strategy *M* is not always the best. For example, if the number of kits is highly limited when compared with the number of patients, that is, $f \simeq 0$ , then strategy $S_1$ may provide smaller loss values. In addition, in Figures 4 and 5, strategy *M* has a wider range of loss value than strategy $S_1$, which may result in a larger loss in some specific situations. This wideness of the loss value range is observable, especially in small medical institutions in group C.

In Figure 6, we mentioned that the loss function is at a minimum value in specific cases. The existence condition for these minimum values is a substantial problem because this situation enables our strategy to achieve the most efficient use of kits. In addition, if a minimum value exists, we can spare some unused kits using our strategy that can then be used in the case of an unexpected patient surge, although an excess reserve could be beneficial in reality, contrary to the loss function defined in Eq. (2).

Some limitations exist in this preliminary study. First, while the SIR predictions have a good fit with the model for the number of patients, the SIR parameters are determined empirically as in Eq. (4). These parameters are derived from patient statistics for the seasonal influenza in Kitami City. Although the model was suitable for the prediction of seasonal influenza in the same city, it would not work for the pandemic influenza and emerging diseases like COVID-19. In such cases, there are no past data that can be applied, and other mechanisms must be used.

Second, while this paper utilised the SIR model for prediction, other alternatives exist. The SIR model usually fits the real data, but this is not always the case. For example, when a single epidemic season has two peaks, as can happen in real life, the SIR model is not sufficient. We need to find other models and adopt them to this kind of data. For example, probabilistic state-space modelling with dynamic Bayesian forecasting has been reported to improve the forecasting of numbers ^12^. Another possibility is to construct a regional model that incorporates the data at all the medical institutions involved, and the relationship among them, such as geographical distance.

Third, we used weekly data from the reported statistics for prediction and strategy. However, in the situation where the number of patients rises quickly, more frequent updates on the strategy might be important. An important future task is to determine how to implement a more dynamic strategy, such as a daily strategy. Also, for simplicity, we assumed a single susceptible group and a single infectious disease. However, the literature has discussed situations where multiple diseases, such as different types of influenza, interfere with each other^13^, and younger and older groups exhibit different characteristics during epidemics^14^. These issues raise other opportunities to revise the strategies.

Lastly, while we assumed that there was no interaction of the diagnosis with interventional outcomes, such as the death toll, it is possible that the choice of the proposed strategies had a non-negligible effect on those critical figures. A new analysis regarding this interaction would contribute to a better understanding of the resource problems and result in a more effective response to epidemic situations.

## Conclusion

In this study, we investigated a resource allocation problem in clinical examination that arises when the demands surpass the available stocks. Although some assumptions may seem unrealistic, the problem is based on a real problem that occurred at the university health center for budgetary reasons. This can also occur when the supply chain is damaged or a resource shortage occurs due to excessive demands, for example, in a pandemic. Previous literature in the domain has focused on the distribution of preventive vaccinations and remedial resources, and thus, this study has addressed a novel aspect of the management of infectious diseases, although the situation is rare.

The clinical implication of the allocation strategies differs, depending on the type of examinations. In the examination for a definitive diagnosis, the allocation strategy affects the workflow of patient management, and thus, the impact is significant. However, in screening tests not for definitive diagnostic purpose but for population estimates or physicians’ diagnostic accuracy evaluation purposes, the examination does not influence patient management. In any case, examinations in the first-come first-served basis can easily exhaust the resources and would result in sampling bias. Additionally, if the presence of testing capacity motivates individuals to seek medical service, the proposed method, which prevents resource depletion, has a clear advantage over the first-come first-served approach. The proposed method is considered meaningful as a method of fairness that uses random sampling of limited resources in a certain period. It is beyond the scope of this study to determine how the presence of such an approach will change treatment outcomes and spread of infectious diseases in a community, but it is worth considering as our future work.

This work makes three contributions to the literature. First, we provided a discussion on the principles that should satisfy the desired strategy for the distribution of diagnostic kits. Second, we pursued an appropriate definition of the loss function to measure the performance and correctness of the strategies. Lastly, we illustrated that, in this distribution problem, a prediction-based strategy outperforms other simple strategies. In strategy *M*, we incorporated the prediction of the number of patients based on an SIR model, while other strategies used no prediction ($S_1$) or only the prediction of term length ($S_2$). Our results show that the strategy using SIR-based prediction was more efficient than the other two strategies that did not use prediction.

For future work, the problem must be generalised to accommodate other infectious agents with different properties and eliminate the presupposition that the simulated period spans an entire epidemic season. For clinical implementation of the approach, physicians need to decide the go or no-go by the prediction model and random numbers. Because the procedure is extremely complex to calculate by hand, a handy smartphone application or Excel spreadsheet is indispensable for the approach to be deployable. Although rapid testing is not significantly invasive for patients, experimental interventions in the allocation of healthcare resources involve ethical issues. Accordingly, we are running the institutional review board process for a clinical research that utilises one of such application. We envision that these preliminary results will help blaze a trail further in this problem domain.

## Supplementary information

Supplementary Information.
