# Supplementary material for: Strategies for the efficient use of diagnostic resource under constraints: a model-based study on overflow of patients and insufficient diagnostic kits
Source: Sci Rep. 2020 Nov 26;10:20740. doi: 10.1038/s41598-020-77468-2 (PMC7692522; doi:10.1038/s41598-020-77468-2)
Supplement: Supplementary file 1 — Supplementary Information. [file 41598_2020_77468_MOESM1_ESM.pdf]

# Supplementary material:
## Strategies for the efficient use of diagnostic resource under constraints: A model-based study on overflow of patients and insufficient diagnostic kits

**Naoshi Tsuchida[1], Fumihiko Nakamura[2], Kazunori Matsuda[2], Takafumi Saikawa[3], and Takashi Okumura[2,*]**

[1]Hokkaido University, School of Medicine, Sapporo, 060-8638, Japan
[2]Kitami Institute of Technology, Kitami, 090-8507, Japan
[3]Nagoya University, Graduate School of Mathematics, Nagoya, 464-0814, Japan
[*]tokumura@mail.kitami-it.ac.jp

## Appendix SIR Model

### A.1 Description

The susceptible–infected–recovered (SIR) model is one of the simplest models in epidemics[1–3]. It divides the population into three "compartments," that is, the susceptible, infected, and recovered (including the dead). The population of each compartment is denoted as $S = S(t)$, $I = I(t)$, and $R = R(t)$, respectively, whose dynamics is presumptively deterministic and governed by the ordinary differential equations as follows:

$$dS/dt = -\beta SI, \quad dI/dt = \beta SI - \gamma I, \quad dR/dt = \gamma I. \tag{1}$$

The total population $N = S + I + R$ is preserved along $t$ since $dN/dt = 0$. The constant $\beta$ shows the disease spreading power, proportional to both the disease transmission probability per contact and the number of contacts per person. The constant $\gamma$ shows the recovery rate of the infected. The parameter called basic reproduction number is defined as $R_0 = \beta N/\gamma$, showing the number of secondary infection generated by single primary infected. The value is considered specific to the disease. In the case of the influenza, the value of $R_0$ is considered as approximately 2-–3[4]. The parameter $\rho = \gamma/\beta = N/R_0$ is called the threshold because the disease epidemic occurs when $\rho < S_0 = S(0)$.

### A.2 Approximation of the SIR model

Equation (1) has a simple approximated solution. From Eq.(1), we obtain $dI/dS = (dI/dt)/(dS/dt) = -1 + \rho/S$ and $dS/dR = (dS/dt)/(dR/dt) = -S/\rho$. These equations can be integrated, resulting in $I = N - S + \rho \log(S/S_0)$ and $S = S_0 \exp(-R/\rho)$. From these equations, we obtain the ordinary differential equation as for $R$,

$$\frac{dR}{dt} = \gamma(N - R - S_0 \exp(-R/\rho)). \tag{2}$$

By assuming $R \ll \rho$ and applying Taylor's expansion to the exponential function up to the second order, we obtain

$$\frac{dR}{dt} = \gamma \left[ N - S_0 + \left( \frac{S_0}{\rho} - 1 \right) R - \frac{S_0}{2\rho^2} R^2 \right]. \tag{3}$$

This nonlinear equation has the following analytical solution:

$$R(t) = \rho(\varepsilon + p\tau(t)), \quad S(t) = S_0 \exp(-\varepsilon - p\tau(t)), \quad \tau(t) = \tanh(bt + c), \tag{4}$$

where

$$b = \frac{\alpha\gamma}{2}, \quad c = -\tanh^{-1}\left( \frac{S_0/\rho - 1}{\alpha} \right), \quad \varepsilon = 1 - \frac{\rho}{S_0} \simeq 1 - \frac{1}{R_0}, \tag{5}$$

$$\alpha = \left[ \left( \frac{S_0}{\rho} - 1 \right)^2 + \frac{2S_0(N - S_0)}{\rho^2} \right]^{1/2}. \tag{6}$$

We have the weekly-reported number of new influenza cases as the data. This number corresponds to the decreasing amount of the susceptible,

$$-\frac{dS}{dt} = Ae^{-p\tau}(1 - \tau^2), \quad \tau = \tau(t) = \tanh(bt + c) \tag{7}$$

where $A = [(S_0\gamma)/2] \times [e^{-\varepsilon}/(1-\varepsilon)] \times p^2$ and $p = \alpha(1-\varepsilon)$. Utilising the parameters appearing in Eq. (5), (6), and (7) ($A$, $b$, $c$, and $p$), the original parameters in the SIR ($\beta$, $\gamma$, $R_0$, etc.)) are written as

$$\frac{1}{\alpha} = \frac{1}{p} + \tanh c, \quad \varepsilon = 1 - \frac{p}{\alpha}, \quad \gamma = \frac{2b}{\alpha}, \quad S_0 = \frac{2Ae^{\varepsilon}(1-\varepsilon)}{\gamma p^2}, \quad \beta = \frac{\gamma}{(1-\varepsilon)S_0}, \quad \frac{1}{R_0} = 1 - \varepsilon. \tag{8}$$

### A.3 Fitting to the data

As introduced in Section 2.1, Figure 1displays the fitting curve using the SIR model, showing that the SIR curve captures both the early and peak stages of the epidemic. In Table 2, we show the basic reproduction number $R_0$, which basically ranges within that in the preceding studies. To evaluate the fitting, we calculated the absolute error of the fitting standardised by its peak scale $\sum_{t=1}^{T} |P_t^R - (-dS/dt)|/AT$ for the data. The values are 0.212 (for 2018) and 0.166 (for 2017), and the standard deviations are 0.233 (for 2018) and 0.123 (for 2017). These values means that the fitting error is around 10–20% on average.

Note that the SIR model is a macroscopic model, which represents an entire population, while our prediction targets an individual medical institution. However, despite the difference, the model fits well with the observed number of patients at the microscopic level, and therefore, we used the SIR model for simplicity.

Figure 2presents the fitting curve changes over time. The curves are unstable before the peak stage (up to the 10–15th week) but become stable after the peak stage. This shows the difficulty in determining the epidemic scale before the peak. This modelling difficulty is not the issue specific to the method of this study but is common in this field[5,6].

## References


**1.** Anderson, R. M., Anderson, B. & May, R. M. *Infectious diseases of humans: dynamics and control* (Oxford university press, 1992).

**2.** Hethcote, H. W. Three basic epidemiological models. In *Applied mathematical ecology*, 119–144 (Springer, 1989).

**3.** Minami, N., Mizuno, K. & Minami, R. Modelling infectious disease epidemic in a population consisting of individuals with different contact rates. In *The Hiyoshi review of the natural science*, vol. 53, 23–44 (Keio gijuku daigaku Hiyoshi kiyo kanko iinkai, 2013).

**4.** Mills, C. E., Robins, J. M. & Lipsitch, M. Transmissibility of 1918 pandemic influenza. *Nature* **432**, 904–906 (2004).

**5.** Bertozzi, A. L., Franco, E., Mohler, G., Short, M. B. & Sledge, D. The challenges of modeling and forecasting the spread of covid-19. *arXiv preprint arXiv:2004.04741* (2020).

**6.** Jewell, N. P., Lewnard, J. A. & Jewell, B. L. Predictive mathematical models of the covid-19 pandemic: Underlying principles and value of projections. *Jama* **323**, 1893–1894 (2020).
